# Identification and Verification of Mitochondria‐Related Diagnostic Markers of Spinal Cord Injury by WGCNA and Machine Learning

**DOI:** 10.1155/bn/1749750

**Published:** 2026-03-04

**Authors:** Haifeng Chen, Wei Cai, Yunpeng Zhang, Xiaoming Tang, Jian Dai, Yao Li, Jian Ma

**Affiliations:** ^1^ Department of Orthopedic, The Affiliated Huaian No. 1 People′s Hospital of Nanjing Medical University, Huaian, Jiangsu, China, njmu.edu.cn

**Keywords:** bioinformatics, immune response, machine learning, mitochondria, spinal cord injury

## Abstract

**Background:**

Spinal cord injury (SCI) significantly impacts patients, with mitochondrial dysfunction playing a critical role in its pathology. Identifying mitochondria‐related genes may offer new therapeutic and prognostic insights.

**Methods:**

RNA sequencing data from the GEO database were analyzed to identify differentially expressed genes (DEGs). Functional enrichment analyses were conducted, and weighted gene coexpression network analysis (WGCNA) alongside machine learning algorithms was used to identify key mitochondria‐related genes. Immune infiltration was assessed using the EPIC algorithm, and single‐cell RNA sequencing (scRNA‐seq) data were analyzed for cellular diversity.

**Results:**

A total of 2566 upregulated and 2634 downregulated genes were identified in SCI versus control samples. GO and KEGG enrichment analyses revealed these DEGs were primarily involved in oxidative stress, mitochondrial function, and immune pathways, including necroptosis and T cell receptor signaling. Then, 1578 genes with the strongest correlation to SCI were selected by WGCNA. By integrating DEGs, WGCNA module genes, and mitochondria‐related genes, 76 candidate genes were obtained and used to construct a PPI network. Six hub genes (NDUFB3, SLC25A24, SLC25A40, GSTZ1, MAOA, and MRPL12) were identified by machine learning, all showing strong diagnostic potential (AUC > 0.77). Immune infiltration analysis indicated reduced B and T cell infiltration and increased macrophage activity in SCI samples. scRNA‐seq analysis further revealed higher expression of NDUFB3 in dendritic cells and MAOA in pro‐B cells, suggesting their involvement in immune regulation and mitochondrial dysfunction.

**Conclusion:**

These six genes represent potential biomarkers and therapeutic targets for SCI, providing insights into its molecular mechanisms and immune response.

## 1. Introduction

Spinal cord injury (SCI) is a severe neurological condition that significantly impacts patients′ quality of life, often resulting from trauma, disease, or congenital defects [1, 2]. SCI not only leads to motor, sensory, and autonomic dysfunction but can also be accompanied by serious complications such as infections, deep vein thrombosis, and cardiovascular diseases [3, 4]. Current treatment methods for SCI are varied, including pharmacological interventions, surgical procedures, and rehabilitation therapies [5]. However, these traditional approaches show notable differences in effectiveness and prognostic evaluation, often failing to target the specific pathological mechanisms of individual patients [6, 7]. Therefore, the development of targeted therapeutic strategies, particularly those aimed at precise interventions based on injury‐related biomarkers, has become a vital direction in SCI research.

Mitochondria play a crucial role in cellular energy metabolism, calcium homeostasis, oxidative stress response, and apoptosis [8]. In recent years, an increasing number of studies have highlighted the significance of mitochondria in SCI [8–10]. Changes in the expression of mitochondria‐related genes are closely associated with neuronal survival and functional recovery following SCI [11]. Furthermore, research has indicated that mitochondrial dysfunction is related to the prognosis of various neurodegenerative diseases, such as Alzheimer′s disease and Parkinson′s disease, suggesting that mitochondria serve not only as a component of disease pathology but also as important prognostic indicators [12–14]. Although SCI is initiated by an acute mechanical insult, its subsequent progression shares several pathophysiological features with chronic neurodegenerative diseases, including oxidative stress, neuroinflammation, metabolic imbalance, and progressive neuronal loss, all of which are closely linked to mitochondrial dysfunction [15]. However, compared with neurodegenerative diseases, the prognostic significance and transcriptomic landscape of mitochondria‐related alterations in human SCI remain far less explored.

Based on the above background, this article is aimed at exploring how to construct a prognostic model for SCI based on mitochondria‐related genes. By analyzing the expression characteristics of mitochondrial genes and their roles in SCI, we hope to offer effective biomarkers for clinical prognostic evaluation. This research not only aids in understanding the pathophysiological mechanisms following SCI but also has the potential to promote the development of personalized medicine.

## 2. Methods

### 2.1. Data Acquisition

Bulk transcriptomic data (GSE151371, 38 SCI and 20 control samples) and scRNA‐seq data (GSE158892, 2 SCI samples) were downloaded from the GEO database (http://www.ncbi.nlm.nih.gov/geo/) [16]. In addition, a list of mitochondria‐related genes (*n* = 1136) was retrieved from GeneCards for downstream integration analyses (https://www.genecards.org).

### 2.2. Identification of Differentially Expressed Genes (DEGs)

DEGs between normal and SCI samples in the GSE151371 dataset were identified using the limma package in R. Moderated *t*‐tests with Benjamini–Hochberg multiple testing correction were applied, and genes with an adjusted *p* value (false discovery rate, FDR) < 0.05 and |*f* *o* *l* *d* *c* *h* *a* *n* *g* *e*| ≥ 1.5 were considered differentially expressed. Visualization of the DEGs was performed with a volcano plot generated using the ggplot2 package.

### 2.3. Gene Ontology (GO) and Kyoto Encyclopedia of Genes and Genomes (KEGG) Enrichment Analysis

GO and KEGG enrichment analyses were conducted to investigate the functional roles of the DEGs. GO terms included biological processes (BPs), cellular components (CCs), and molecular functions (MFs). The analyses were executed with the R packages “clusterProfiler” and “org.Hs.eg.db” with Benjamini–Hochberg correction for multiple testing, and terms with an adjusted *p* value (FDR) < 0.05 were regarded as significantly enriched. Besides, results were visualized using the “Goplot” package.

### 2.4. Weighted Gene Coexpression Network Analysis (WGCNA)

WGCNA was applied to identify gene modules related to SCI. To enhance the accuracy of the analysis, samples were clustered to exclude outliers. The optimal soft threshold was selected to ensure a network structure approximating a scale‐free distribution. Gene coexpression modules were constructed using this soft threshold, and a hierarchical clustering tree was generated. The minimum gene module size was set to 50, with a module merging threshold of 0.25. Correlation analysis was performed to identify key modules related to SCI, and genes within the most relevant modules were selected for hub gene identification.

### 2.5. The Identification of Hub Genes Using Machine Learning

Three machine learning algorithms—random forest (RF), support vector machine (SVM), and LASSO Cox—were used to identify characteristic genes associated with SCI. Genes identified by all three methods were considered hub genes.

### 2.6. Immune Infiltration Analysis

The estimation of proportions of immune and cancer cells (EPIC) algorithm within the “IOBR” package was employed to assess immune cell infiltration. The EPIC method estimates the proportions of five immune cell types (B cells, CD4^+^ T cells, CD8^+^ T cells, macrophages, and NK cells), cancer‐associated fibroblasts (CAFs), endothelial cells, and uncharacterized cells (primarily cancer cells) from bulk gene expression data. This approach enables more accurate deconvolution of immune cell infiltration proportions in solid tumor analyses, effectively mitigating biases introduced by the high expression background of tumor cells.

### 2.7. scRNA‐seq Analysis

Preprocessing and filtering of scRNA‐seq data were carried out using the Seurat package. At first, processed gene expression matrices were downloaded from GEO and imported into Seurat to construct a Seurat object and subsequently subjected to rigorous quality control. In detail, quality control criteria included nFeature_RNA > 500, nCount_RNA between 1000 and 20,000, and percent.mt < 20*%*. After normalization using the NormalizeData, principal component analysis (PCA) was conducted to identify significant principal components, followed by t‐distributed stochastic neighbor embedding (t‐SNE) analysis to delineate cell clusters. Finally, we used the SingleR package with the HumanPrimaryCellAtlasData reference to obtain initial labels for each cluster based on transcriptomic similarity. These automated annotations were then manually cross‐checked using canonical marker genes.

### 2.8. Regulatory Network Construction and Drug Sensitivity Prediction

miRNAs regulating hub genes were identified through the miRWalk database. For each hub gene, we extracted miRNAs supported by at least one experimentally validated or high‐confidence prediction source within miRWalk. The resulting gene‐specific miRNA lists were intersected using a Venn diagram to identify miRNAs that cotargeted multiple hub genes. The final set of eight overlapping miRNAs was used to construct a putative miRNA–mRNA regulatory network in Cytoscape.

### 2.9. Statistical Analysis

All statistical analyses were performed using R software (Version 4.0.3) and SPSS. The diagnostic value of hub genes was evaluated with receiver operating characteristic (ROC) curves. Unless otherwise specified, all statistical tests were two‐sided. Pearson correlation analysis was performed on log2‐transformed gene expression data under the assumption of approximate normality, and statistical significance was set at *p* < 0.05.

## 3. Results

### 3.1. The Identification of DEGs

**Figure 1:**
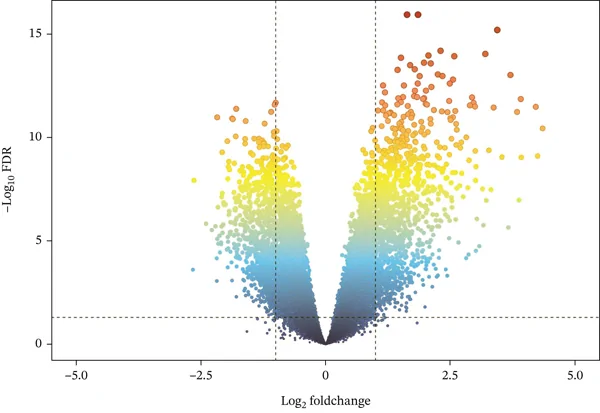
The volcano diagram showed the differentially expressed genes in the SCI group compared with the control group.

At first, we calculated the fold change of all genes between normal and SCI groups. The results are shown in Figure 1, and it can be seen that 2566 genes were upregulated in the SCI group compared with the control group, while 2634 genes were downregulated.

### 3.2. The GO and KEGG Enrichment Analysis of DEGs

Then, we explored the potential mechanism of DEGs in the development of SCI. As shown in Figure 2a, the DEGs mainly existed in the vesicle, endomembrane system, and cytosol. Besides, in the aspect of MF, the main functions of DEGs included cation binding, catalytic activity, and enzyme binding (Figure 2b). In addition, DEGs were enriched in transport, establishment of localization, and response to stress in the term of BP (Figure 2c). Additionally, DEGs were mainly involved in necroptosis, T cell receptor signaling pathway, and NOD‐like receptor signaling pathway (Figure 2d). Previous studies have demonstrated that these pathways were closely linked to mitochondrial reactive oxygen species production and immune regulation after SCI [17–19]. It suggested that mitochondrial dysfunction may promote inflammatory cell death and innate immune activation while simultaneously impairing T cell–related immune responses.

Figure 2The enrichment analysis of DEGs using GO and KEGG. (a) The enrichment results of DEGs in CC of GO analysis. (b) The enrichment results of DEGs in MF of GO analysis. (c) The enrichment results of DEGs in BP of GO analysis. (d) The enrichment results of DEGs in KEGG analysis.

**Figure:**
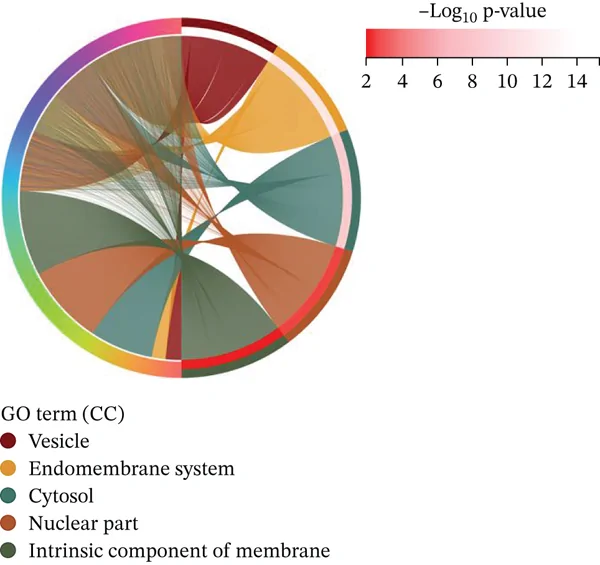
(a)

**Figure:**
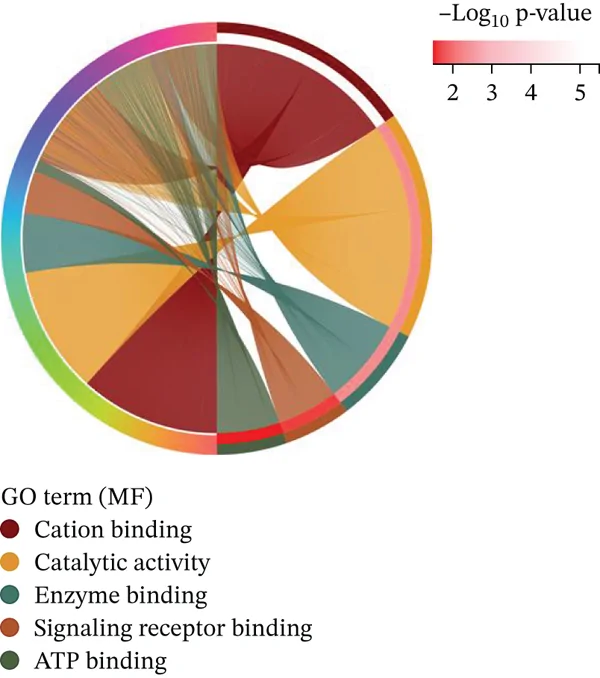
(b)

**Figure:**
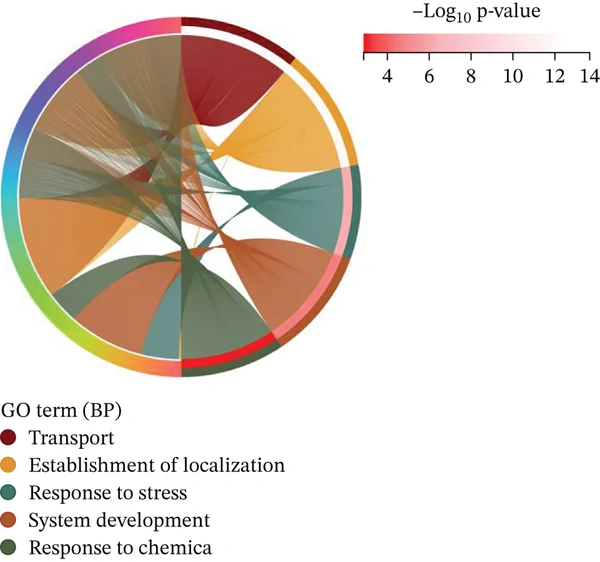
(c)

**Figure:**
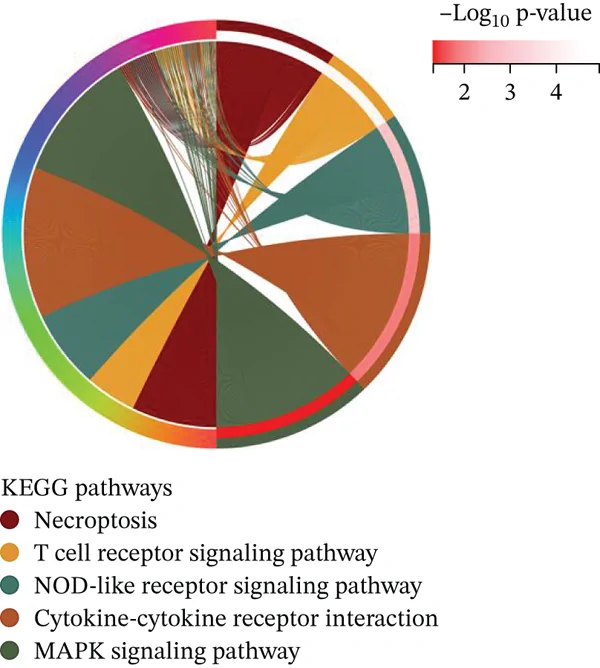
(d)

### 3.3. WGCNA

Next, we applied the WGCNA to select the genes related to the development of SCI. At first, a soft threshold *β* = 18 was utilized to establish a scale‐free network, leading to the identification of 11 distinct modules (Figures 3a, 3b, 3c, and 3d). Among them, the lightgreen and orangered modules, respectively, showed the strongest positive and negative correlations with SCI status in the module–trait heatmap, which indicated that these genes likely represented the group most closely associated with the onset and progression of SCI. Thus, 1578 genes in the lightgreen (correlation coefficient = 0.76, *p* < 0.001) and orangered modules (correlation coefficient = −0.75, *p* < 0.001) were selected for further analysis (Figure 3e).

Figure 3Coexpression modules were identified and characterized by WGCNA in SCI. (a, b) The scale independence and the mean connectivity of the WGCNA. (c) The eigengene adjacency heatmap and clustering of the 11 distinct modules. (d) Heatmap of eigengene adjacency showing distances between module eigengenes. (e) Module–trait relationships showing correlation between module eigengenes and two groups (control and SCI).

**Figure:**
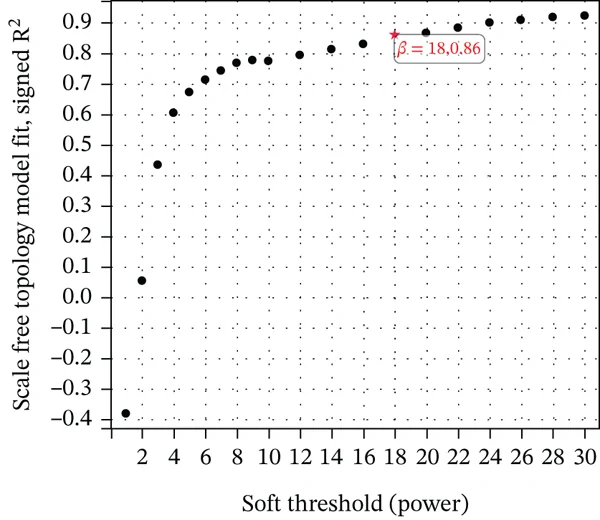
(a)

**Figure:**
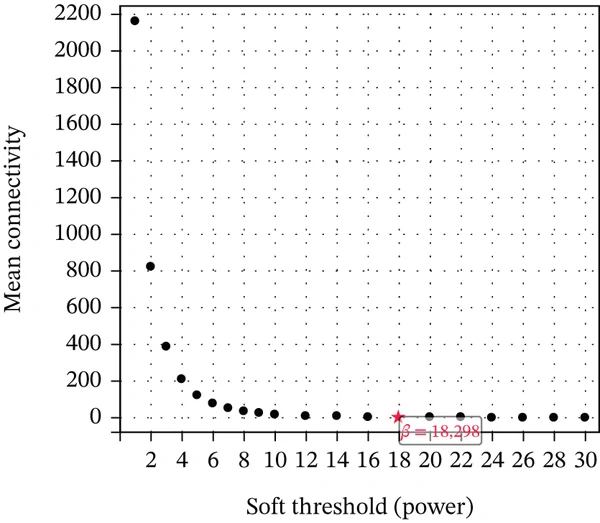
(b)

**Figure:**
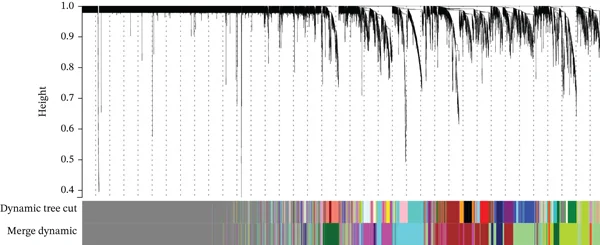
(c)

**Figure:**
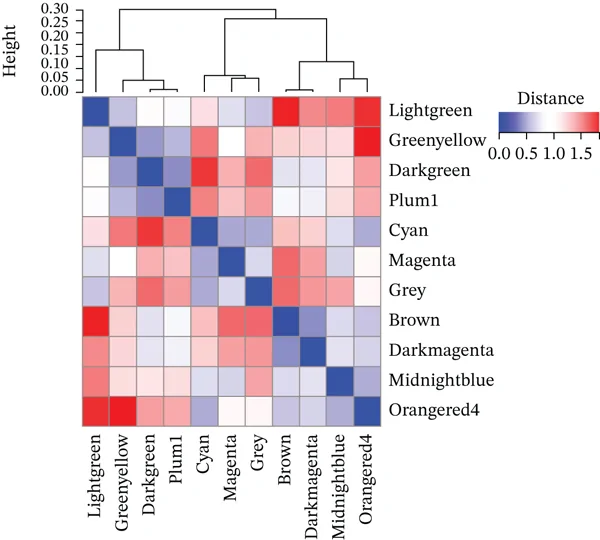
(d)

**Figure:**
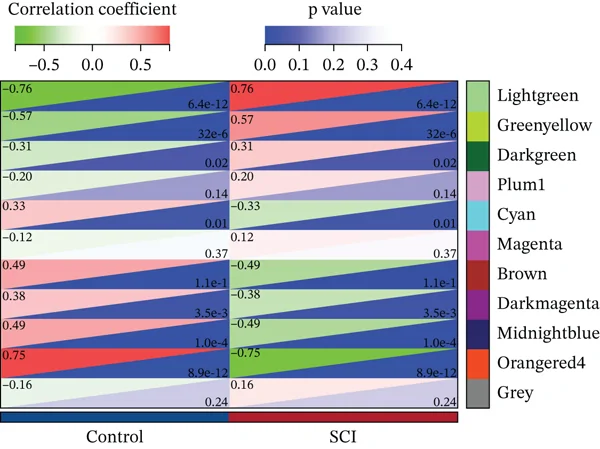
(e)

### 3.4. The PPI Network Among Gene MRGs Related to the Development of SCI

Next, according to the above results, 1339 genes overlapped with the DEG list among the 1578 genes in these two modules. To move beyond global differential expression patterns, we intersected DEGs with SCI‐associated WGCNA modules and mitochondria‐related gene sets. This integrative strategy reduced thousands of DEGs to 76 candidate genes that were simultaneously differentially expressed, coexpressed in SCI‐relevant modules, and functionally linked to mitochondrial biology. These genes were considered more likely to represent core molecular alterations underlying SCI‐related mitochondrial dysfunction rather than secondary or nonspecific transcriptional changes (Figure 4a). Besides, the PPI network of these genes is visualized in Figure 4b.

Figure 4The correlation between MRGs related to the development of SCI. (a) The Venn diagram shows the MRGs related to the development of SCI. (b) The PPI network among MRGs.

**Figure:**
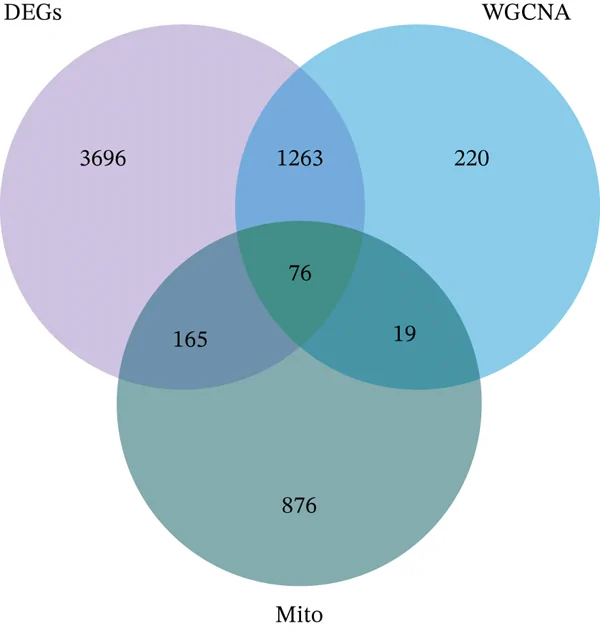
(a)

**Figure:**
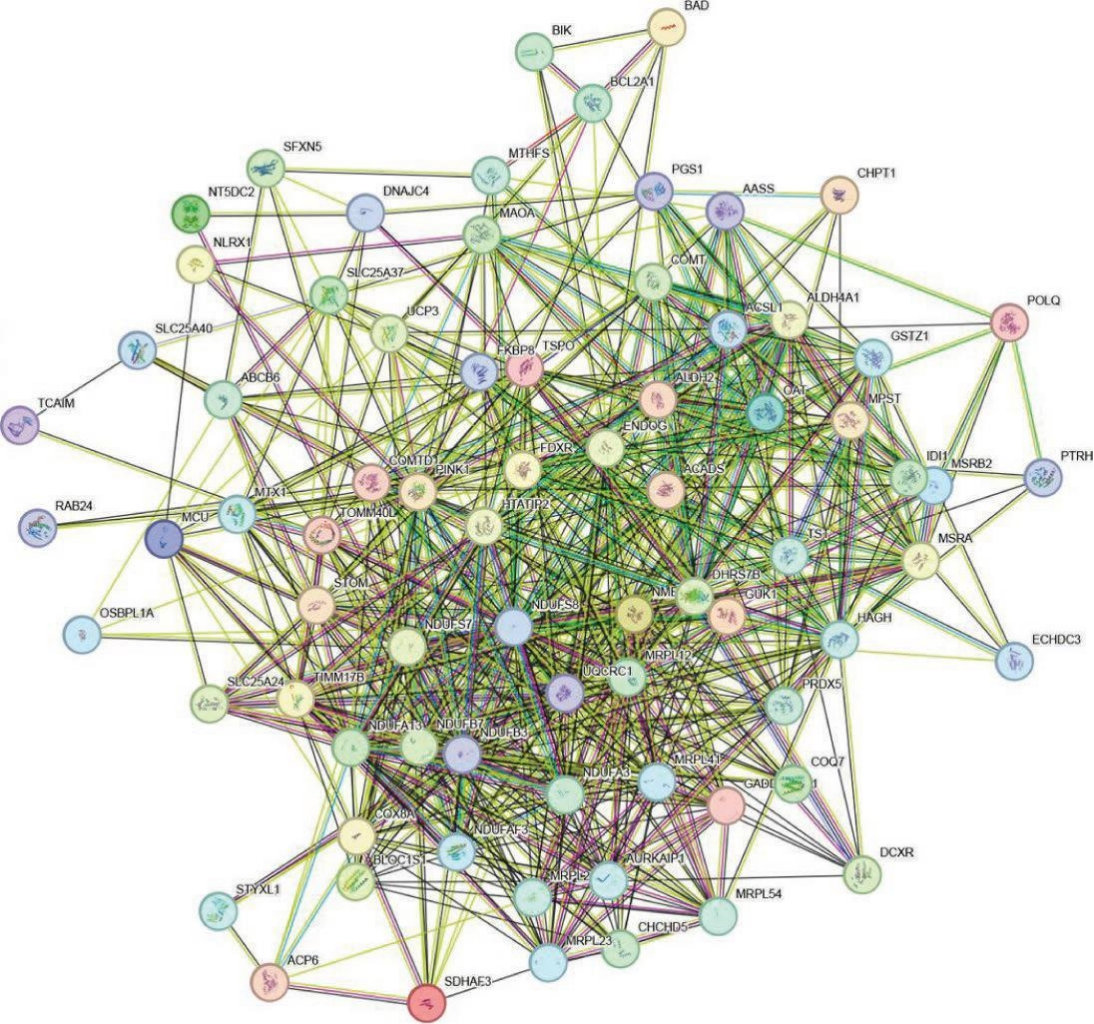
(b)

### 3.5. The Identification of Hub Genes

Then, to further determine the hub genes, three machine learning algorithms were performed. Based on the SVM algorithm, there is highest accuracy and lowest error when 34 characters were screened out (Figure 5a,b). According to the RF algorithms, the top 10 genes with the highest importance were selected (Figure 5c). In the LASSO regression analysis, an optimal lambda value was determined after 10 cross‐validations, and 14 key genes were identified (Figure 5d,e). Then, the six overlapping genes of three algorithms were considered as hub features (Figure 5f).

Figure 5The hub genes were screened out through machine learning. (a, b) Selection of characteristic genes through SVM algorithm. (c) The top 10 genes with the highest importance. (d, e) Ten‐time cross‐validation in the LASSO model and LASSO coefficient profiles. (f) The Venn diagram illustrates overlapping genes of the LASSO, RF, and SVM algorithms.

**Figure:**
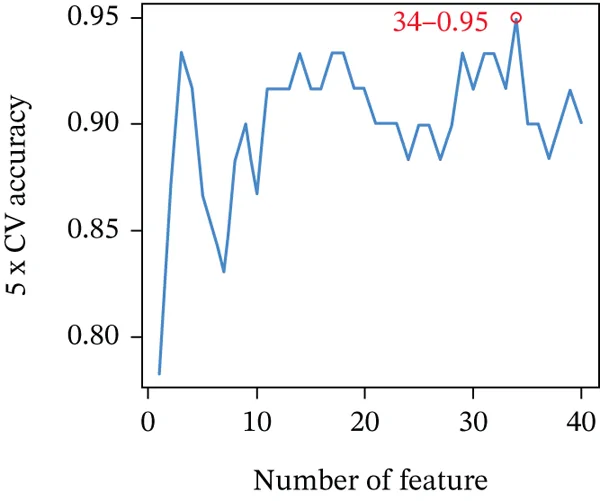
(a)

**Figure:**
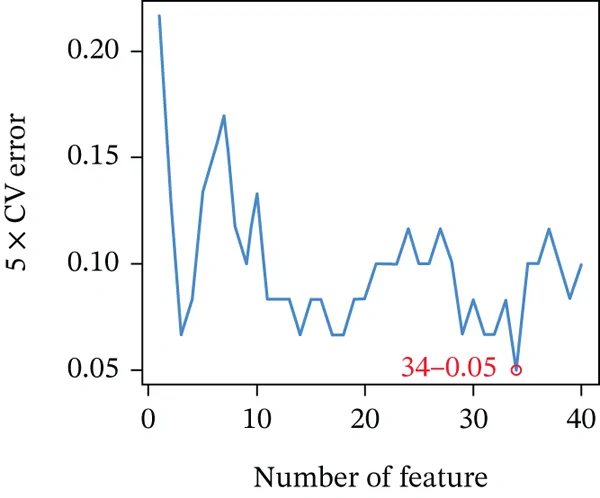
(b)

**Figure:**
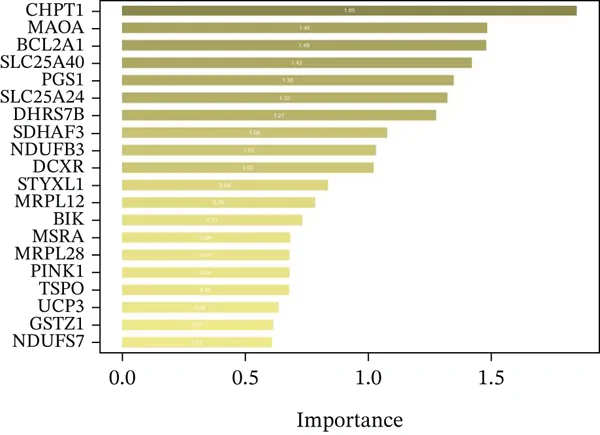
(c)

**Figure:**
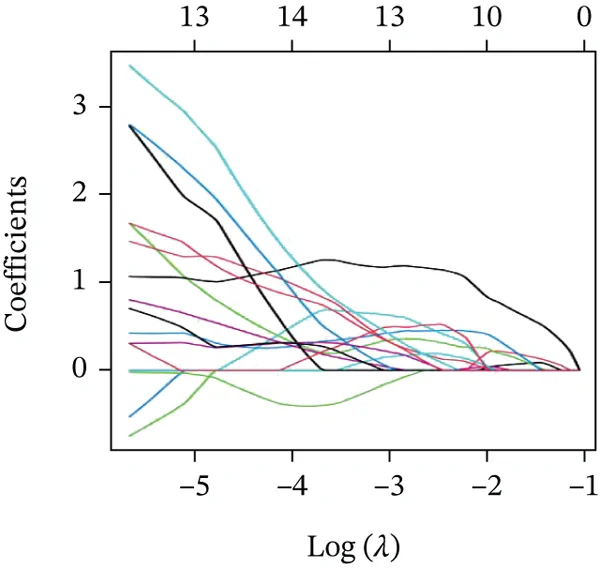
(d)

**Figure:**
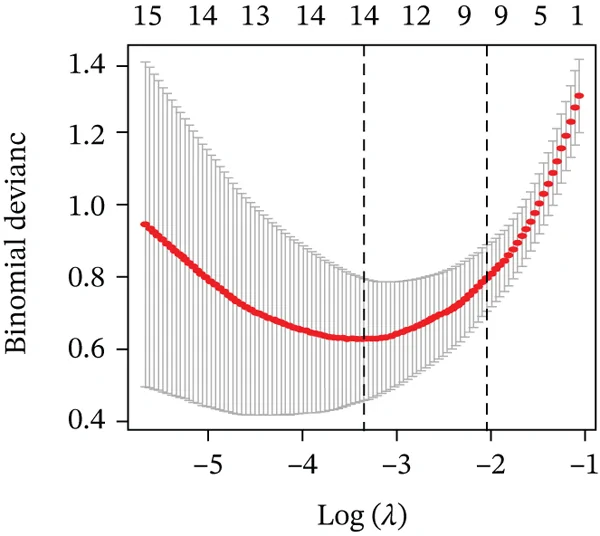
(e)

**Figure:**
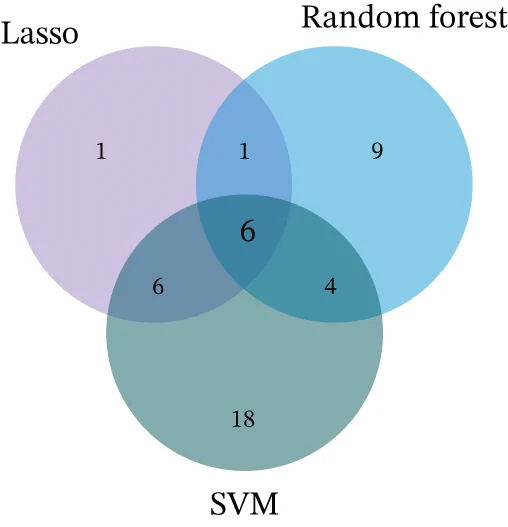
(f)

### 3.6. The Expression and Diagnostic Value of Hub Genes

To explore the role of hub genes in the development of SCI, we first analyzed their expression. As shown in Figure 6a, it was obvious that all hub genes were upregulated in the SCI group compared with the control group. Besides, the ROC curve based on the GSE151371 dataset exhibited that six hub genes had good predictive value for the diagnosis of SCI, and the AUC values were all greater than 0.77 (Figures 6b, 6c, 6d, 6e, 6f, and 6g).

Figure 6The expression and ROC curve of hub genes. (a) The expression of six hub genes in SCI and normal groups. Blue indicates the control group, and red indicates the SCI group. The ROC curve of (b) SLC25A24, (c) GSTZ1, (d) MRPL12, (e) NDUFB3, (f) SLC25A40, and (g) MAOA.

**Figure:**
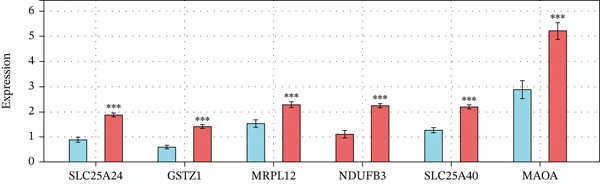
(a)

**Figure:**
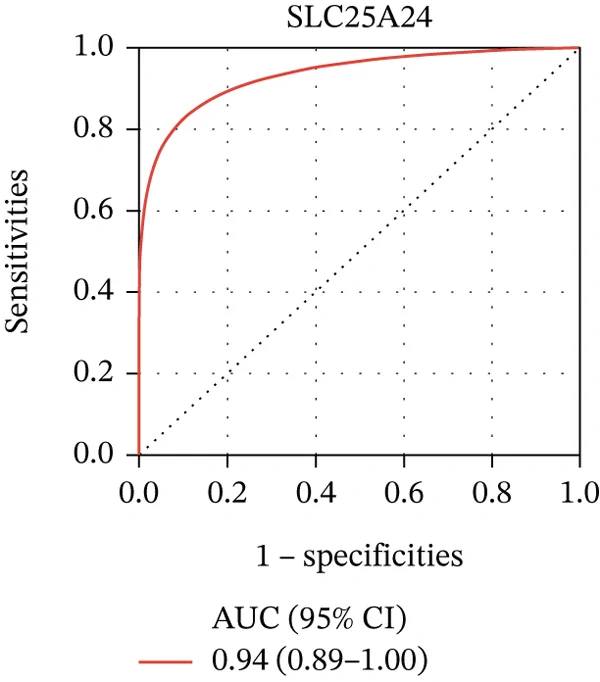
(b)

**Figure:**
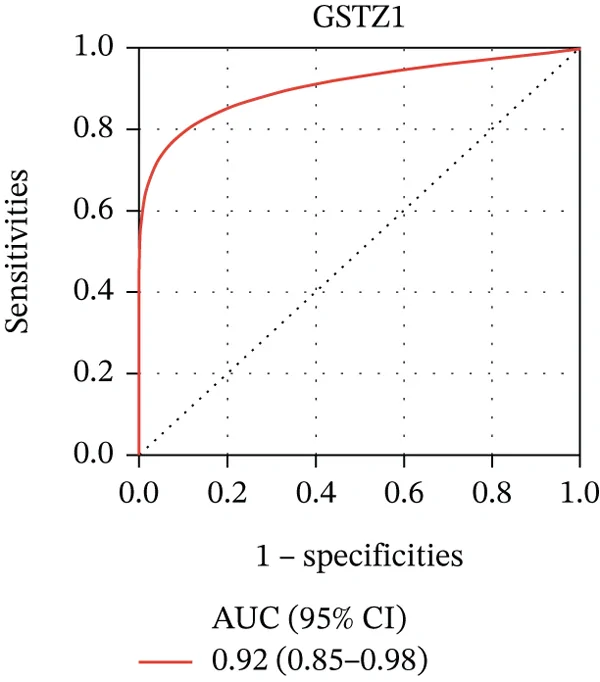
(c)

**Figure:**
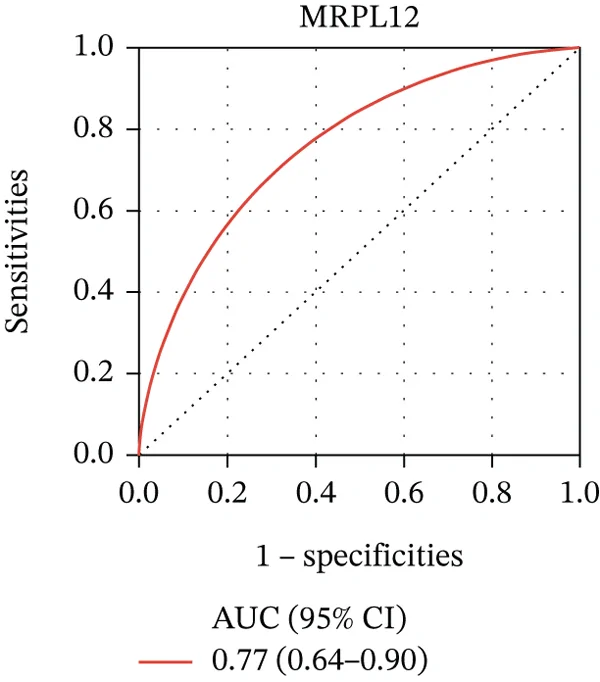
(d)

**Figure:**
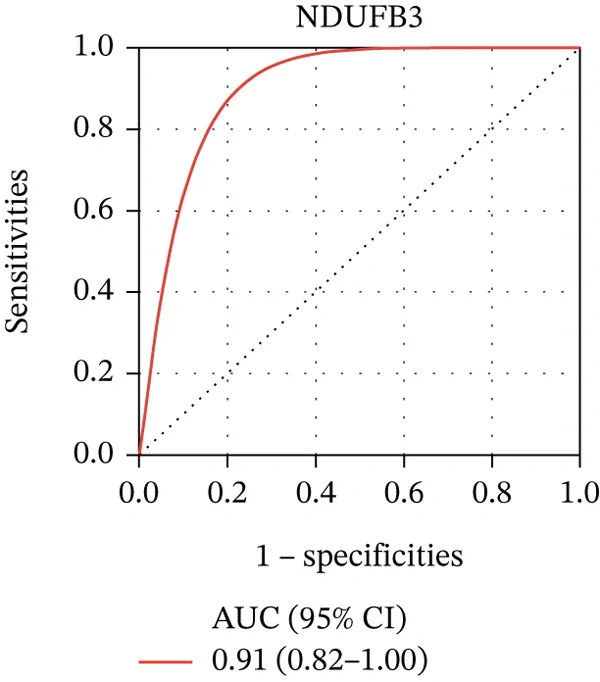
(e)

**Figure:**
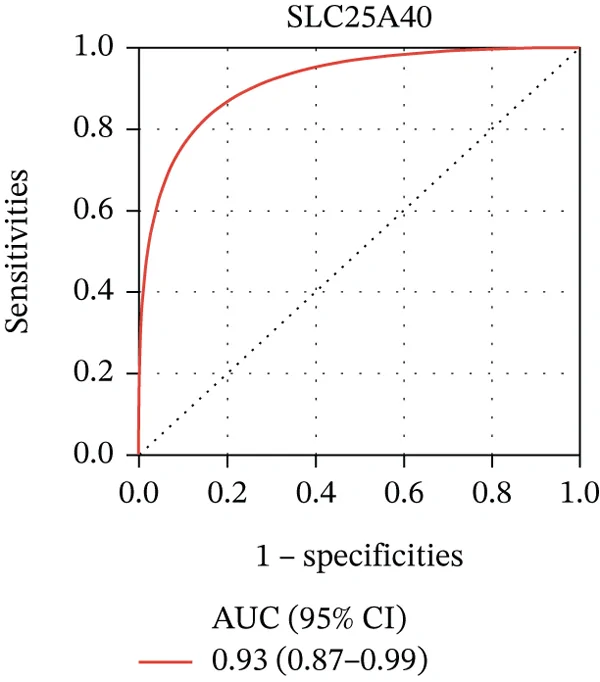
(f)

**Figure:**
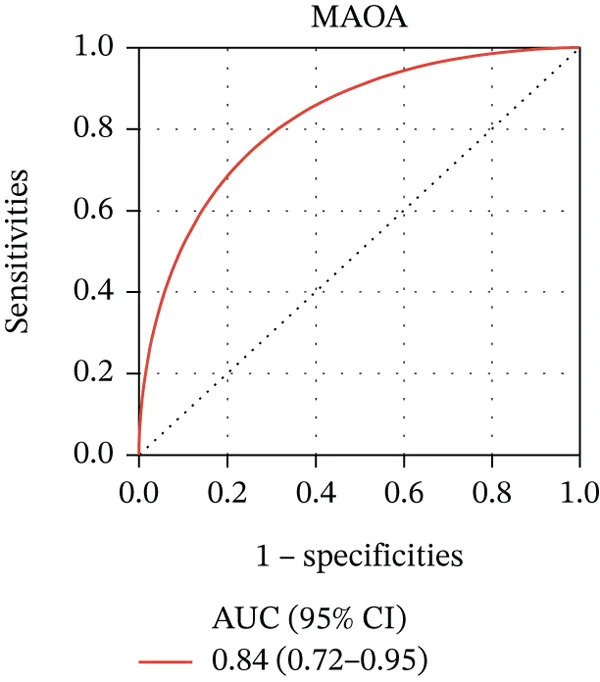
(g)

### 3.7. The Immune Infiltration Analysis

The KEGG results indicated that immune response may play an important role in the development of SCI. Thus, we calculated the immune infiltration level of different immune cells in the normal and SCI samples through EPIC algorithm. As shown in Figure 7a, it was obvious that the infiltration levels of B cells, CD4^+^ T cells, and CD8^+^ T cells were all significantly decreased in SCI compared with the normal group, while macrophage levels were increased. Besides, the correlation analysis exhibited that B cell levels were negatively related to the MAOA, and CD4^+^ T cell levels were negatively related to SLC25A24, GSTZ1, NDUFB3, and SLC25A40. In addition, CD8^+^ T cell levels were negatively related to SLC25A24, SLC25A40, and MAOA, and macrophage levels were positively related to SLC25A24 and MAOA (Figure 7b). These results suggested that these hub genes may be involved in shaping the immune microenvironment after SCI.

Figure 7The immune infiltration and correlation analysis. (a) The infiltration levels of immune cells through the EPIC algorithm. (b) The correlation between immune cells and hub genes.

**Figure:**
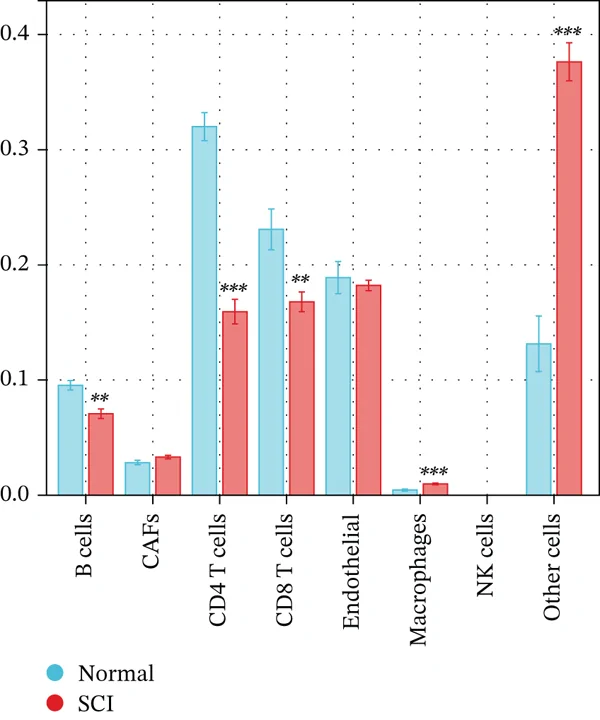
(a)

**Figure:**
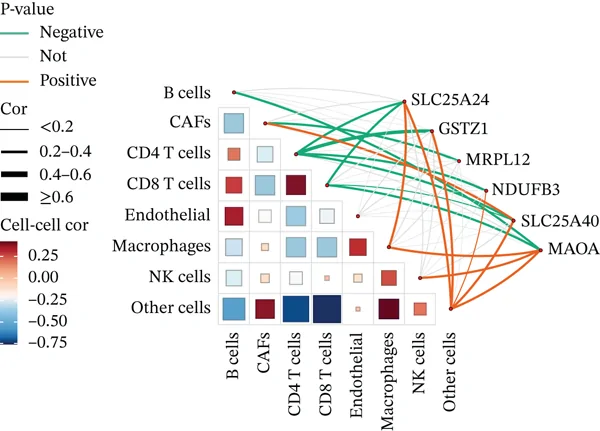
(b)

### 3.8. The scRNA Analysis Based on GEO Database

In this study, two SCI samples were used for the scRNA‐seq based on the GSE158892 dataset. After the quantity control treatment and expression normalization, the dimension of cells was reduced by PCA, and 11 PCs were determined (Figure 8a). Then, all cells were separated into 23 clusters through the t‐SNE algorithm, and cell subpopulations were annotated by SingleR (Figure 8b,c). Clusters 10 and 14 were DC cells, and Cluster 8 was macrophage. Furthermore, it can be seen that NDUFB3 expression was significantly higher in DC cells than in other cells, while MAOA expression was significantly higher in CD34^+^ pro‐B cells than in other cells.

Figure 8The determination of cell type in SCI based on two samples. (a) The PCA dimension reduction of 1–20 PCs. (b) Twenty‐three clusters were identified using the t‐SNE algorithm. (c) Six cell subtypes were annotated by SingleR. (d) The expression of six hub genes in six cell types.

**Figure:**
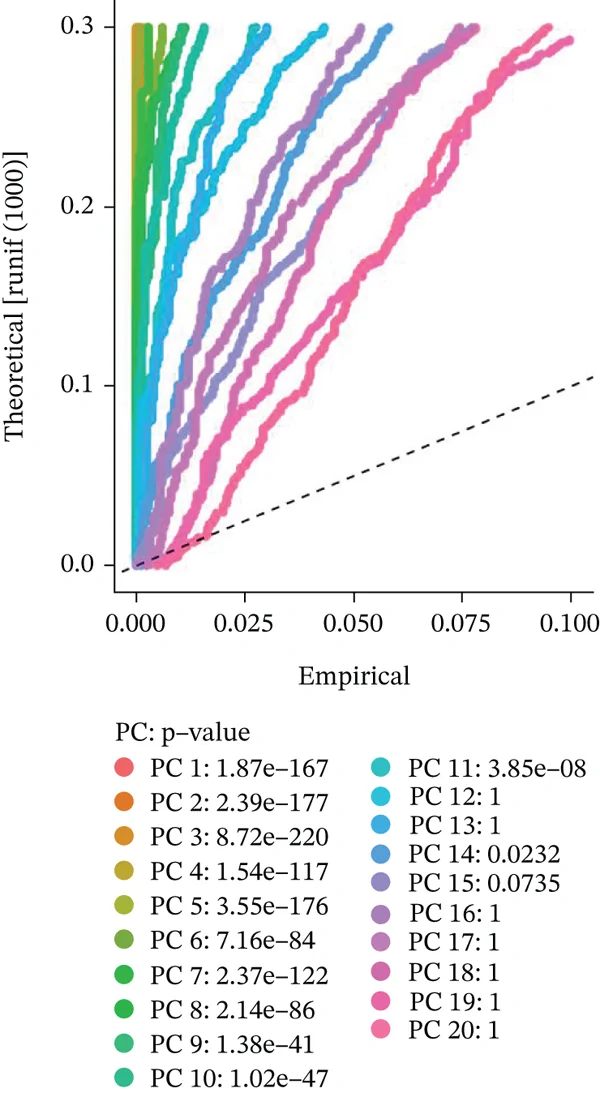
(a)

**Figure:**
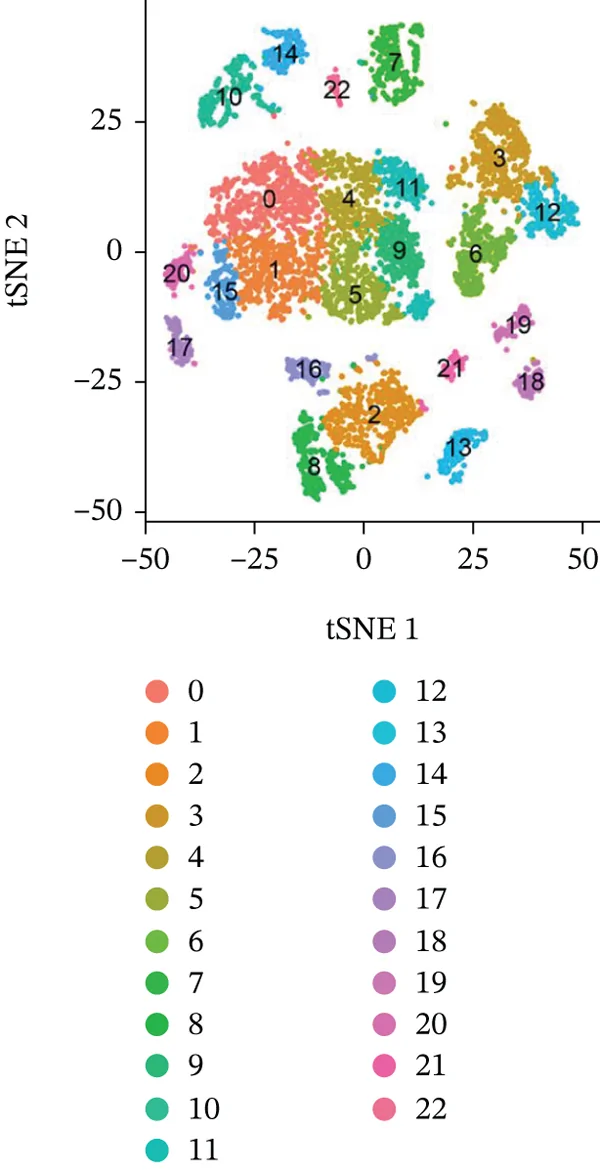
(b)

**Figure:**
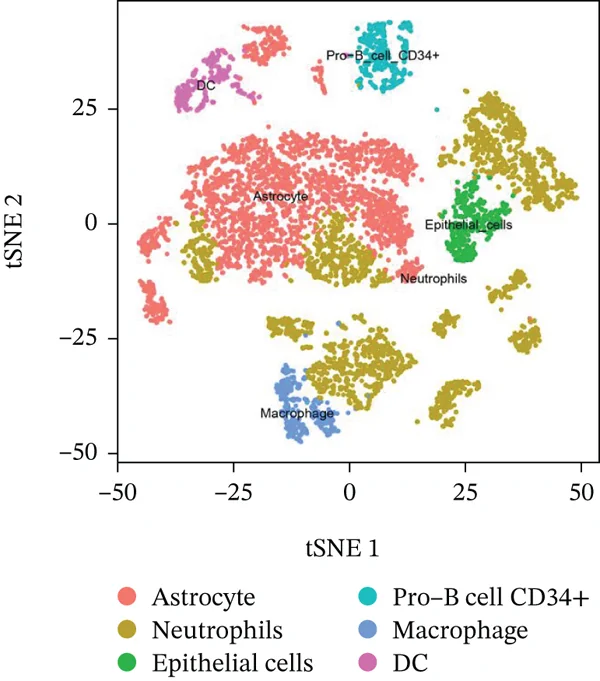
(c)

**Figure:**
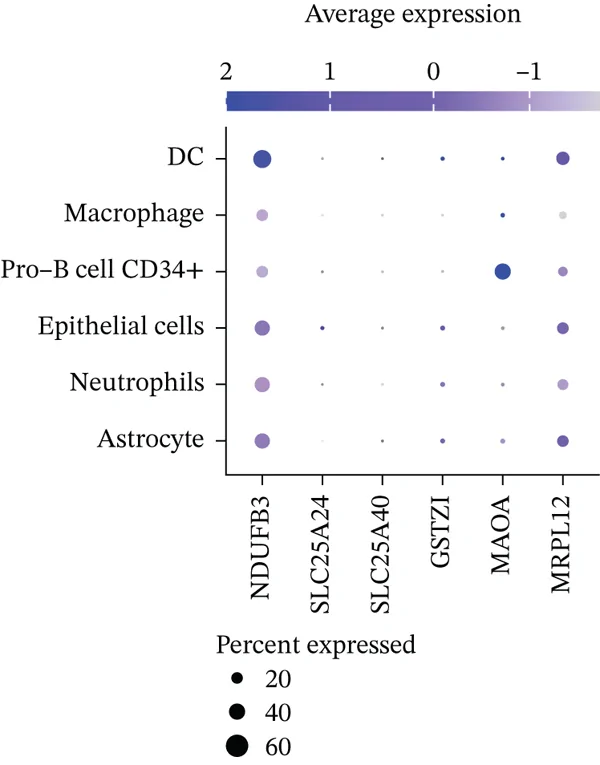
(d)

### 3.9. The miRNA–mRNA Regulatory Network

Finally, we tried to explore the regulatory network of hub genes. For each hub gene, we extracted miRNAs supported by at least one experimentally validated or high‐confidence prediction source within miRWalk. The resulting gene‐specific miRNA lists were intersected using a Venn diagram to identify miRNAs that cotargeted multiple hub genes (Figure 9a). The final set of eight overlapping miRNAs was used to construct a putative miRNA–mRNA regulatory network in Cytoscape (Figure 9b).

Figure 9The construction of the miRNA–mRNA regulatory network. (a) The Venn diagram shows the miRNA targeted hub genes. (b) The miRNA–mRNA regulatory network based on the correlation between miRNA and mRNA.

**Figure:**
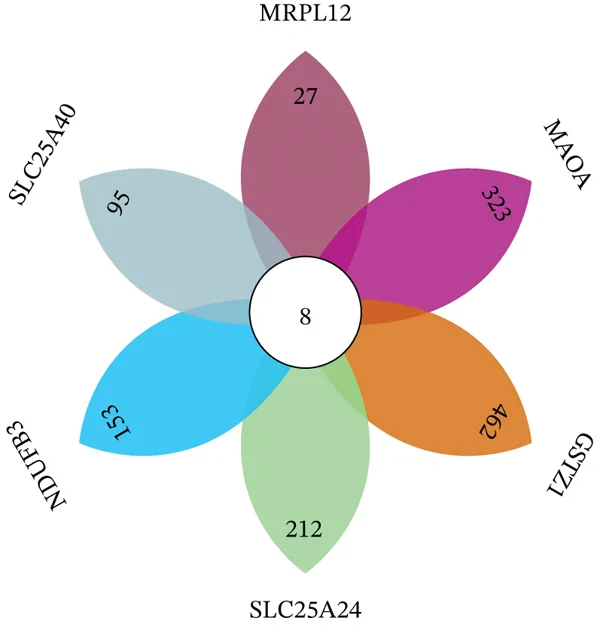
(a)

**Figure:**
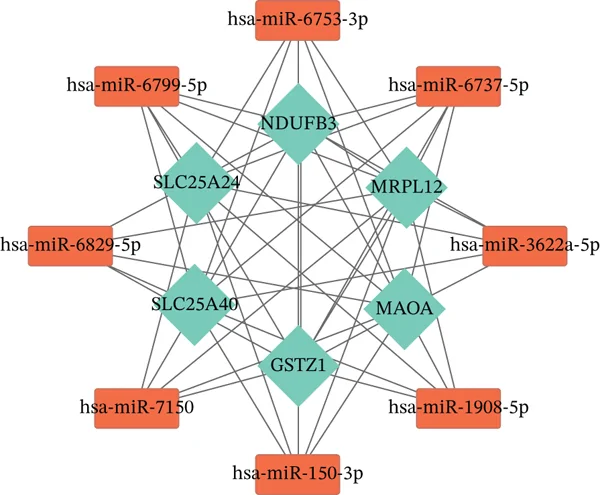
(b)

## 4. Discussion

SCI is associated with substantial morbidity, and secondary injury processes such as inflammation and cell death critically influence neurological recovery [1]. Using integrated bulk and single‐cell transcriptomic analyses, we identified mitochondria‐related candidate biomarkers and observed immune cell alterations that may reflect postinjury immune remodeling. In this study, we performed bioinformatics analysis on the transcriptomic data of SCI patients and control samples using the GEO database. Through differential expression analysis, WGCNA, and machine learning, we identified six mitochondria‐related genes (NDUFB3, SLC25A24, SLC25A40, GSTZ1, MAOA, and MRPL12). Through ROC curve analysis, we found that all six genes have excellent diagnostic value.

NDUFB3 is a part of mitochondrial respiratory chain complex I, responsible for electron transport and ATP production [20]. Zhu et al. found that overexpression of NDUFB3 can reduce mitochondrial ROS levels in thyroid cancer and inhibit tumor growth, suggesting that NDUFB3 may induce ROS production in mitochondria [21]. Our study shows that NDUFB3 is significantly elevated in SCI, which indicates that ROS levels may also be significantly increased. Although elevated ROS levels can inhibit disease progression by promoting apoptosis, stimulating immune effector cells, and suppressing immunoregulatory cells, excessive ROS may damage immune cells, thereby suppressing immune responses [22]. Additionally, Wang et al. found that high ROS levels lead to inflammation and macrophage activation [23]. Our results also revealed that B cell and T cell infiltration levels were significantly reduced in the SCI group, while macrophages significantly increased. This may be because macrophages are the primary producers of ROS [24].

SLC25A24 and SLC25A40 are members of the SLC family, responsible for transporting calcium ions and other metabolites across the mitochondrial membrane [25]. Although studies have found that SLC25A24 is overexpressed in various cancers and SLC25A40 is significantly elevated in leukemia, their potential functions remain unclear [26, 27]. In our study, we found that both SLC25A24 and SLC25A40 were significantly upregulated in SCI, and they were significantly negatively correlated with CD4^+^ T and CD8^+^ T cell infiltration levels. This suggests that the two may play important roles in regulating immune cell responses.

GSTZ1 is a member of the glutathione transferase (GST) superfamily [28]. Research has shown that GSTZ1 is downregulated in hepatocellular carcinoma (HCC) and inhibits the progression of HCC by downregulating the Wnt/*β*‐catenin signaling pathway [29]. Abnormal expression of GSTZ1 can lead to the inactivation of DCA dechlorination, thereby affecting DCA metabolism [30]. DCA has shown excellent results in the clinical treatment of SCI [31]. In addition, numerous studies have suggested that GSTZ1 is involved in metabolic reprogramming processes such as the TCA cycle and tyrosine metabolism [32]. Our results show that GSTZ1 is significantly overexpressed in SCI, suggesting that GSTZ1 may promote SCI development by regulating energy metabolism.

MAOA is an enzyme responsible for degrading monoamine neurotransmitters, which play crucial roles in both the central and peripheral nervous systems [33]. Previous studies have shown that these neurotransmitters participate in immune cell regulation during inflammation and stress responses [34]. Further research revealed that MAOA can promote TAM immunosuppressive polarization by upregulating oxidative stress [35]. Similarly, we found that MAOA expression levels and macrophage infiltration levels were significantly elevated in SCI, with a significant positive correlation between MAOA and macrophages. It should be noted that the observed relationships between hub genes and immune cell subsets are correlational and do not imply causality. Nevertheless, the positive association between MAOA expression and macrophage infiltration, together with previous reports linking MAOA to immunosuppressive macrophage polarization, raises the possibility that MAOA may modulate macrophage function and oxidative stress in SCI. Similarly, altered expression of mitochondrial transporters SLC25A24 and SLC25A40 might affect T cell survival and effector function by influencing mitochondrial calcium handling and energy metabolism. These hypotheses require validation in dedicated mechanistic studies.

MRPL12, as a key component of the mitochondrial ribosome, plays a central role in maintaining mitochondrial protein synthesis and oxidative phosphorylation functions [36]. Its critical role in metabolic reprogramming and energy production makes it an important factor in regulating tumor cell proliferation and survival [37]. Additionally, Ji et al. found that MRPL12 can protect cells from apoptosis by stabilizing mitochondrial membrane homeostasis [38]. These findings suggest that MRPL12 may be involved in disease progression through its regulation of energy metabolism and apoptosis.

However, there are some limitations in this study. First, the analyses relied entirely on publicly available datasets from GEO, which may be affected by batch effects and technical heterogeneity. Second, the sample sizes, particularly for the scRNA‐seq dataset (two SCI samples), were relatively small, limiting statistical power and generalizability. Third, ROC curves were calculated in the discovery cohort without additional internal cross‐validation or external validation, raising the possibility of overfitting. Fourth, the roles of the identified hub genes and the predicted miRNA–mRNA interactions were not validated experimentally. Finally, cross‐species or independent clinical cohorts were not available for validation. Therefore, our findings should be regarded as hypothesis‐generating and warrant confirmation in larger, prospectively collected datasets and mechanistic studies.

## Author Contributions

Haifeng Chen, Wei Cai, and Yunpeng Zhang contributed to the conception and design. Wei Cai, Xiaoming Tang, Yao Li, and Jian Dai contributed to the collection and assembly of data. Haifeng Chen, Yunpeng Zhang, Xiaoming Tang, and Jian Ma analyzed and interpreted the data. Haifeng Chen, Wei Cai, Yunpeng Zhang, Xiaoming Tang, Jian Dai, and Yao Li contributed equally to this work and should be regarded as co‐first authors.

## Funding

No funding was received for this manuscript.

## Disclosure

All authors wrote and approved the final manuscript.

## Ethics Statement

The Ethics Committee of the Affiliated Huaian No. 1 People′s Hospital of Nanjing Medical University deemed that this research is based on open‐source data, so the need for ethics approval was waived.

## Consent

The authors have nothing to report.

## Conflicts of Interest

The authors declare no conflicts of interest.

## Data Availability

The datasets generated during and/or analyzed during the current study are available from the corresponding author on reasonable request.
